# Recent Advances in Applications of Bioactive Egg Compounds in Nonfood Sectors

**DOI:** 10.3389/fbioe.2021.738993

**Published:** 2021-12-16

**Authors:** Xiaoying Zhang, Brindha Chelliappan, Rajeswari S, Michael Antonysamy

**Affiliations:** ^1^ Chinese-German Joint Laboratory for Natural Product Research, College of Biological Science and Engineering, Shaanxi University of Technology, Hanzhong, China; ^2^ Centre of Molecular and Environmental Biology, University of Minho, Department of Biology, Braga, Portugal; ^3^ Department of Biomedical Sciences, Ontario Veterinary College, University of Guelph, Guelph, ON, Canada; ^4^ Department of Microbiology, PSG College of Arts & Science, Bharathiar University, Coimbatore, India

**Keywords:** hen egg, biomedical resource, market potential, bioactive peptides, nonfood applications

## Abstract

Egg, a highly nutritious food, contains high-quality proteins, vitamins, and minerals. This food has been reported for its potential pharmacological properties, including antibacterial, anti-cancer, anti-inflammatory, angiotensin-converting enzyme (ACE) inhibition, immunomodulatory effects, and use in tissue engineering applications. The significance of eggs and their components in disease prevention and treatment is worth more attention. Eggs not only have been known as a “functional food” to combat diseases and facilitate the promotion of optimal health, but also have numerous industrial applications. The current review focuses on different perceptions and non-food applications of eggs, including cosmetics. The versatility of eggs from an industrial perspective makes them a potential candidate for further exploration of several novel components.

**Systematic Review Registration:** [website], identifier [registration number].

## Highlights

1. Biologically active ingredients of hen eggs are widely used in medicine/veterinary medicine.
2. Egg ingredients have been widely used in bio-industries.
3. Transgenetic henscan produce eggs with large number of specific proteins/peptides of need.
4. Nonfood uses of hen eggs needs to be highlighted in egg research and egg industry.

## Introduction

**FIGURE 1:**
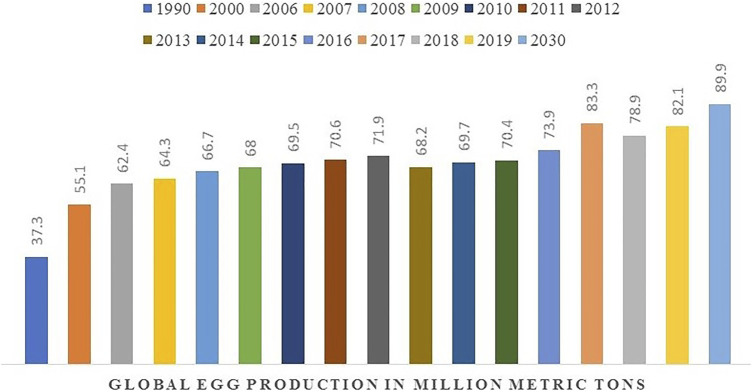
Global egg production rate with expected.

**FIGURE 2:**
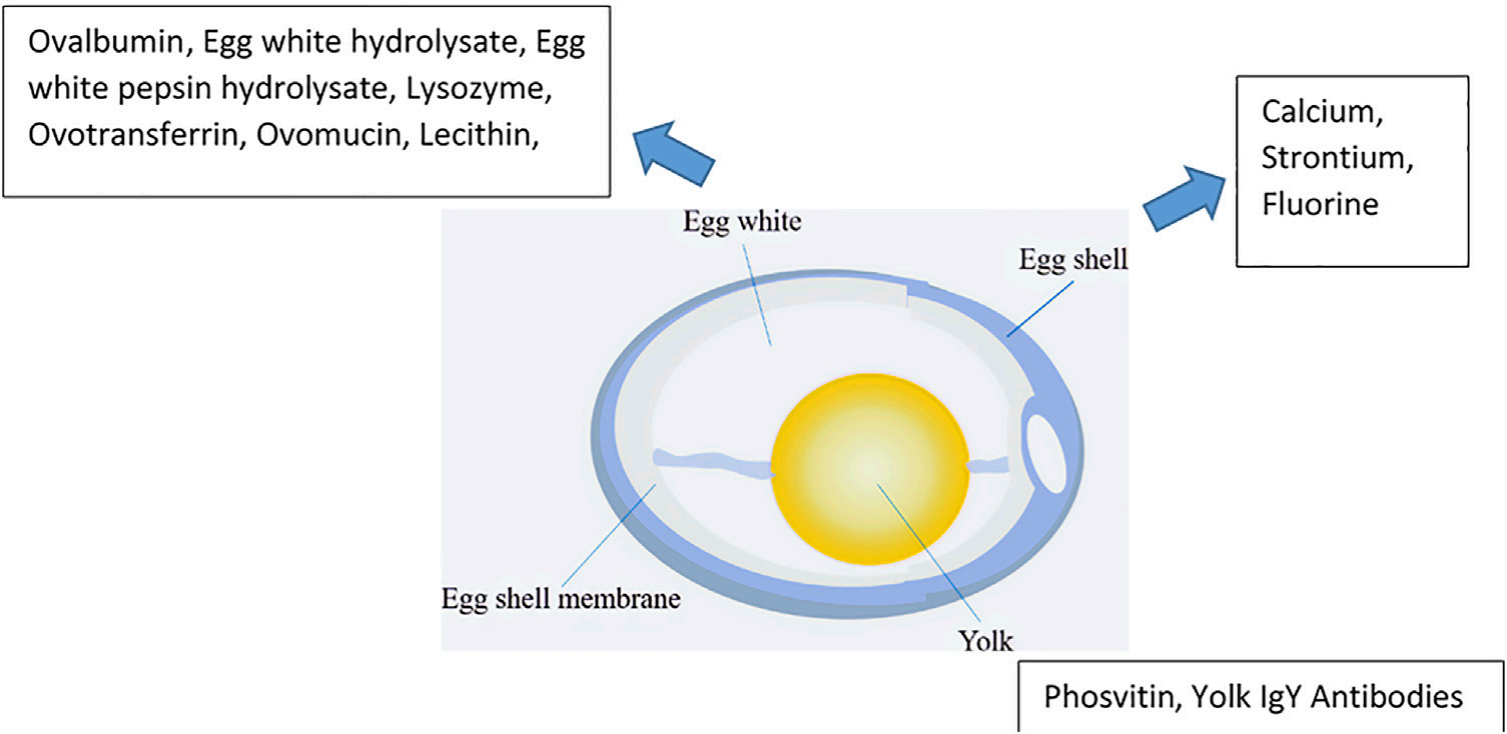
Structure of a hen’s egg projections.

The consumption of eggs, particularly hen eggs, has a long history alongside the development of human civilization. The global egg production was 73.8 million tons in 2016 and is expected to reach 89.9 million tons in 2030, with an average annual increase of 1.6% (Conway, 2012; Portal Statistics, 2018) (Figure 1). The principal components of eggs include the eggshell (ES), egg white (EW), the yolk, and the eggshell membrane (ESM) (Figure 2).

In addition to food consumption, increasing attention has been given to exploring the unique biological values and functions of eggs and their comprehensive applications as a non-food resource in different industrial sectors. This review aimed to summarize and analyze the cost-effectiveness of this essential food product that can make the academic communities and industries explore its potential, its bioactive components, and the potential use of its industrial waste for recycling and development into profitable non-food products (Abeyrathne et al., 2013). In addition, an overall understanding of its bioactive components and their potential in large-scale development for applications in biotechnology, medicine, pharmaceuticals, cosmeceuticals, and nutraceuticals may give rise to future developments in the egg industry (Anton et al., 2006). Extensive studies have been carried out to identify and characterize the biologically active components of hen’s eggs, apart from the products already being produced by industries for various biomedical applications (Kovacs-Nolan and Mine, 2004), for human and veterinary medicine, and other potential applications in non-biological industries (Bhat et al., 2015).

## Bioactive Egg Compounds in Human Medicine

**FIGURE 3:**
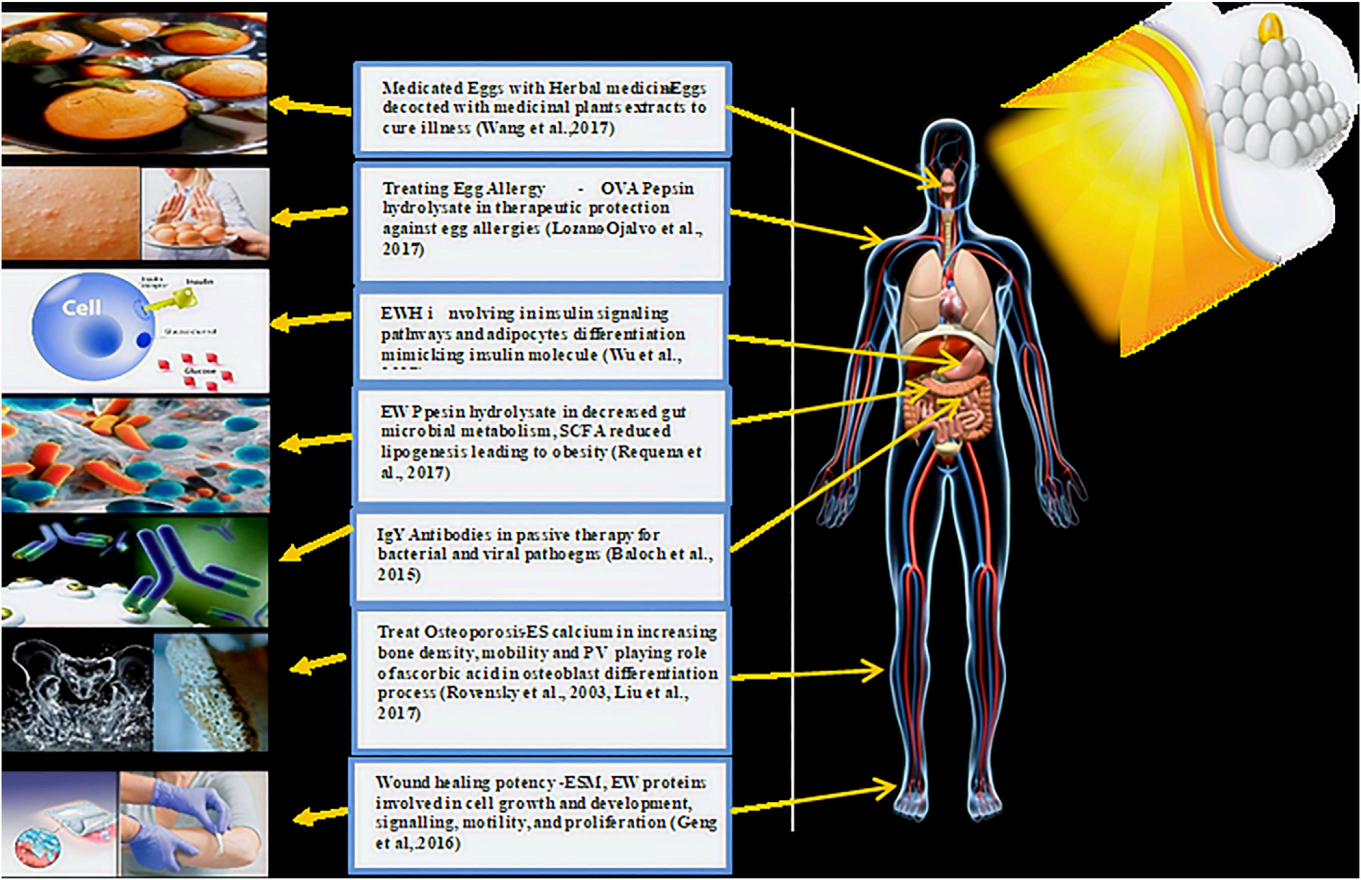
Role of eggs and its bioactive components in human medicine.

Chicken eggs harbor numerous active biological ingredients and have the potential to serve as raw materials in various biomedical sectors and industries (Table 1; Figure 3).

**TABLE 1:** Functional property of Egg components.

Functional characteristics	Hen egg compounds	References
Antibacterial	Lysozyme, Ovalbumin, Ovotransferrin, Ovomucin, Avidin, Phosvitin, IgY	(Valenti et al. (1982); Tsuge et al. (1996); Tsuge et al. (1997); Oguro et al. (2000); Sattar Khan et al. (2000); Horie et al. (2004); Anton et al. (2006); Omana et al. (2010); Moon et al. (2011); Charernsriwilaiwat et al. (2012); Memarpoor-Yazdi et al. (2012); Abeyrathne et al. (2013); Baron et al. (2014); Carrillo et al. (2014); Kim et al. (2014); Marie et al. (2015); Guyot et al. (2016); Zheng et al. (2016); Sun et al. (2017))
Anti-cancer	Lysozyme, Ovomucin, Ovotransferrin, IgY	(Das et al. (1992); Kobayashi et al. (2004); Ibrahim and Kiyono, (2009); Khoozani et al. (2014); Mahanta et al. (2015); Yousr et al. (2017); Lee et al. (2017); Lee, et al. (2017))
Anti-inflammatory	Phospholipids, Lutein/Zeaxanthin, Ovotransferrin, High-density Lipoproteins, Phosvitin	(Wu and Acero-Lopez, (2012); Abeyrathne et al. (2013); Ren et al. (2015); Spillmann et al. (2015); Liao et al. (2016); Sun et al. (2016); Chen et al. (2017); Grootaert et al. (2017); Lee et al. (2017); Lee et al. (2017))
Antioxidants	Ovalbumin, Ovomucin, Ovotransferrin, Lysozyme, Cystatin, Ovoinhibitor, Phosvitin, Phospholipids, Carotenoids, Lutein/Zeaxanthin, Vitamin E, Selenium, Aromatic amino acids, High-density Lipoproteins, Phosvitin	(Ishikawa et al. (2005); Abeyrathne et al. (2013); Liu et al. (2014); Ren et al. (2014); Nimalaratne et al. (2015); Duan et al. (2016); Oladipo and Ibukun, (2017))
Anti-adhesive	Bioactive peptides	(Huopalahti, (2007); Bhat et al. (2015))
Antihypertensive	Bioactive peptides, Lysozyme, Ovomucin, Egg yolk hydrolysates, HDL	(Navab et al. (2011); Reiner et al. (2011); Rao et al. (2012); Eckert et al. (2014); Pokora et al. 2014; Abeyrathne et al. (2015); Eftekhar et al. (2015); Nimalaratne et al. (2015); Nowacki et al. (2017))
Antiviral	IgY, Ovomucin, Ovotransferrin, Bioactive peptides, Lysozyme	(Tsuge et al. (1996); Li et al. (2014))
Immunomodulation	Lysozyme, Ovomucin, Ovalbumin, Ovotransferrin, Bioactive peptides	(Huopalahti, (2007); Kobayashi et al. (2015); Li et al., 2017; Liu et al. (2017); Liu et al., 2017)
Protease inhibition	Cystatin, Ovomucin, Ovomacroglobulin, Ovoinhibitor	(Kovacs-Nolan et al. (2005); Huopalahti, (2007))

### Ovalbumin for the Treatment of Egg Allergy

Allergies to eggs are the second most common food allergy, next only to milk, which could be countered with oral immunotherapy using peptides to avoid atopic reactions and prolonged treatment regimes. Ovalbumin (OVA), a 45-kDa phosphorylated glycoprotein, is responsible for the antigenic and allergenic properties of eggs, comprising 54% w/v of the total egg white proteins. The amino acid sequences that belong to the serpin family of proteinase inhibitors are composed of three genes, *X*, *Y*, and *OVA* (Huopalahti, 2007). It has been reported that the ability of OVA pepsin hydrolysate with intact OVA to treat egg allergies in BALB/c mice expressed better therapeutic protection against egg allergies by inducing regulatory cells (Tregs) and upregulating the expression of TGF-β, IL-10, IL-17, Foxp3, and RORγt in the intestinal tissues (Lozano-Ojalvo et al., 2017). Thus, oral formulations containing OVA pepsin hydrolysates can be employed in the future for infants and children to reduce the risk of egg allergies.

### Egg Components in Improving Osteoporosis

The eggshell is a rich source of calcium in addition to strontium, fluorine, and other minerals, and is a potential industrial raw material for use in applications such as bone metabolism. The positive effects of calcium on bone and cartilage in experiments involving piglets, female rats, and postmenopausal women, are evidenced by a reduction in pain, osteoporation, increased bone density, and mobility (Rovenský et al., 2003). Shell hydroxyapatite mimicking human bones and teeth can be used to prepare bio-composite materials for human soft tissues, bone implantations, dental fixtures in various forms of powders, and porous blocks (Quina et al., 2016). In addition, eggshell nano-additives can be employed in soft drinks to prevent tooth erosion (Khoozani et al., 2014).

Egg yolk phosvitin, a highly phosphorylated protein naturally found in nature, plays a vital role in the osteoblast differentiation process, similar to ascorbic acid. Real-time PCR analysis of cultured mouse osteoblastic MC3T3-E1 cells treated with ascorbic acid and phosvitin revealed a similar expression of osteogenic gene markers, including collagen type I, osteocalcin, runt-related transcription factor 2, and bone morphogenetic protein-2 (Liu et al., 2017). Phosvitin can effectively play the role of ascorbic acid in the osteoblast differentiation process when the former is unavailable, with immediate applications for individuals who are susceptible to bone loss, providing alternative treatment options for patients with osteoporosis. Industries currently manufacturing artificial bone and dental fixtures can replace them with a natural biological material containing eggshell waste to rapidly improve bone structure formation.

### Insulin Mimetic Property and Metabolic Syndrome

Insulin resistance and inflammation in the adipose tissue increase the risk of metabolic syndrome. Egg white hydrolysate (EWH) exhibits antioxidant, anti-inflammatory, and antihypertensive properties by inhibiting the activity of the angiotensin-converting enzyme (ACE) in the renin-angiotensin system (RAS), which is also involved in insulin signaling pathways and adipocyte differentiation (Martinez et al., 2019). EWH displays insulin-mimetic and sensitizing effects, and a previous study showed the effects of these hydrolysates on insulin signaling in adipocytes (Jahandideh et al., 2017). The high incidence of cardiovascular diseases (CVD) and diabetes have led to huge market demand for insulin (and insulin-like) molecules. In summary, the analysis of the insulin-mimetic property of EWH may aid in the effective prophylaxis and management of metabolic syndrome in the future.

### Obesity Control Concerning the Gut Microbiota

EW pepsin hydrolysates could reduce short-chain fatty acids in association with the gut microbiota to reduce the incidence of obesity-associated complications and dyslipidemia. A reduction in the microbial load in the feces along with high concentrations of short-chain fatty acids (SCFA), lactate, fecal lactate, and ammonium were observed in obese rats treated with 750 mg/kg body weight of EW pepsin hydrolysate in drinking water for 12 days in comparison to the control. The reduction in microbial load (including *Lactobacillus, Enterococcus,* and *Clostridium leptum*), in turn, diminished microbial metabolism as evidenced by a decrease in SCFA levels, which was attributed to the antioxidant and anti-inflammatory properties of EW during lipogenesis (Requena et al., 2017).

### Wound Healing Potency of Egg Compounds

The use of ESM to improve biological and biodegradable matrices has gained attention as a new material for use in biological dressings in the wound regeneration process in split-thickness skin graft donors (Huang et al., 2013), and in nerve regeneration enhancement in the sciatic nerves of rats (Farjah et al., 2013). ESM, with its biodegradability due to proteoglycans, has been successfully used to treat non-healing wounds and burns (Mogoşanu and Grumezescu, 2014). Manufacturing wound dressings from massive amounts of ESM industrial waste may have the market potential for solutions involving chronic wound healing. Similarly, EW proteins displayed proliferative bioactivity, are involved in cell migration, and have rapid wound healing properties (Lee et al., 2013; Geng et al., 2016). A total of 33 proteins have been identified *via* LC-MS/MS in EW, most of which play important roles in cell growth and development, signaling, motility, and proliferation. The bioactivity of these candidate molecules suggests that EW contains essential compounds that contribute to the growth of an embryo before fertilization (Lee et al., 2013).

### IgY Antibodies in Human Medicine

Egg yolk antibody (IgY), as a possible substitute for mammalian antibodies, has been used for the diagnosis, prevention, and treatment of infections caused by bacteria and viruses (Kovacs-Nolan and Mine, 2004; Spillner et al., 2012; Baloch et al., 2014; Thu et al., 2017). The use of IgY in human medicine has gained interest in passive immunotherapeutic clinical conditions, including colitis, influenza, and bacterial and fungal infections (such as those caused by *Clostridium botulinum*, *Staphylococcus aureus*, *Candida albicans*, *Helicobacter pylori*, and *Pseudomonas aeruginosa*) (Horie et al., 2004; Kovacs-Nolan and Mine, 2012). For instance, IgY has been incorporated into toothpaste and mouthwash to reduce the levels of oral *Streptococcus mutans* (Chi et al., 2004). At present, many mature IgY drugs have already entered the market, including *Helicobacter pylori* NCT02721355 and *Pseudomonas aeruginosa* NCT01455675 (Leiva et al., 2020). Clinical trials involving IgY showed encouraging results, catapulting products with mono-specific or mixed IgY formulations into the novel nutraceuticals or health supplements market for use in prophylaxis against several diseases. With the rising consumer demand towards natural products for health concerns, IgY is an emerging pharmaceutical preparation from a functional food that can be used soon (Huopalahti, 2007; Thu et al., 2017).

### Anti-Cancer Effects of Egg Compounds

Lysozyme was the first protein from hen eggs with its sequence known. It can prevent bacterial carcinogenesis and improve the effects of anti-cancer drugs and induce rapid recovery from immune suppression (Zheng et al., 2016). Treatment with a preparation containing self-assembled nanostructured lysozyme particles showed a tumor inhibition rate of 31.53 and a survival extension of 4–5 days in different murine tumor models (Das et al., 1992), as well as an inhibition of breast cancer cell migration (Mahanta et al., 2015). An egg yolk gel filtration fraction (EYGF-33) from EW pepsin and pancreatin hydrolysate displayed anti-proliferative activity in human colon cancer cells (Caco-2), inhibiting cell viability without affecting human colon epithelial normal cells (HCECs) after treatment for 48 h. The viability of cancer cells was suppressed by the initiation of apoptosis upon administration of EW hydrolysates (Yousr et al., 2017). Ovotransferrin (OVT) safeguards the developing embryo with its antioxidant, antimicrobial, anti-cancer, and strong metal chelation properties (Valenti et al., 2012). Functional peptides obtained from OVT hydrolysis possess strong anti-cancer effects against colon and breast cancer cells (Ibrahim and Kiyono, 2009). Peptides from Pramod 278p (OTH-P) and thermolysin (OTH-T) hydrolysis showed anti-cancer activity in human stomach adenocarcinoma cells with <20% cytotoxicity against MRC-5 cells (human normal lung fibroblasts) (Lee et al., 2017; Lee et al., 2017).

Biologically active peptides from OVT and lysozyme can be employed in cancer treatments, including for colon and breast cancer, since they have reduced cytotoxicity against normal cells as well as interfere with cancer cell progression.

### Antioxidant Activity of Egg Bioactive Peptides

Various compounds obtained from eggs, including albumin, OVT hydrolysates, and phosvitin complexes exhibit antioxidant and anti-inflammatory properties. The peptide (NTDGSTDYGILQINSR) produced by pepsin, *via* trypsin hydrolysis, or lysozyme from albumen showed antioxidant and antimicrobial activity against both Gram-positive bacteria (*Leuconostoc mesenteroides*) and Gram-negative bacteria (*Escherichia coli*) (Memarpoor-Yazdi et al., 2012). Even with a limited number of studies exploring the use of antioxidants from animal sources, evidence has shown that the antioxidant property of egg proteins decreases after cooking, while gastrointestinal tract (GIT) digestion increases the antioxidant potential of hydrolyzed peptides obtained from egg proteins. The antioxidant activity of minute amounts of lutein and zeaxanthin contributes to ocular health improvement by preventing macular degeneration and the development of cataracts (Handelman et al., 1999). Cystatin, an inhibitor of cysteine proteinases with antibacterial properties, can also modulate NO• synthesis and protect brain neurons from oxidative damage, demonstrating its potential activity as an antioxidant (Nimalaratne and Wu, 2015).

### ACE Inhibition and Drug Candidates Against Cardiovascular Diseases and Oxidative Stress

Hypertension and its associated complications can be minimized by the inhibition of ACE in the RAS. ACE inhibitory peptides (Met-Lys-Arg, Arg-Gly-Tyr, and Val-Ala-Trp) from the enzymatic hydrolysis of HEW lysozyme exhibited high inhibitory activity (Rao et al., 2012), but more comprehensive studies on the mechanism of their absorption from the intestinal walls to the bloodstream need to be conducted to determine their pharmacologic potential.

ACE inhibitory peptides from the multienzyme hydrolysate of ovomucoid (OVM) with pepsin, trypsin, and alcalase can lower the blood pressure (Abeyrathne et al., 2015). A pentapeptide from OVM pepsin hydrolysate, Trp-Asn-Trp-Ala-Asp (WNWAD), has an extraordinary oxygen radical absorption capacity in HEK-293 cells (Abeyrathne et al., 2015).

High-density lipoprotein (HDL) is associated with an increased risk of CVD, as evidenced by epidemiological studies, and protects against atherosclerotic CVD (Reiner et al., 2011) by promoting reverse cholesterol transport (Navab et al., 2011). A decreased incidence of atherosclerotic plaques with increased plasma HDL levels and reverse cholesterol transport from tissues to the liver have been observed from experimental studies on rats, suggesting that HDL is an effective anti-atherosclerotic agent for high-risk populations of patients with CVD (Eftekhar et al., 2015).

Lecithin esterified from fatty acids significantly reduces blood pressure and lowers the serum levels of inflammatory factors. Serum levels of oxidative stress markers such as nitro-tyrosine and heart rate were lowered by approximately 30%–34% in both hypertensive and normotensive animals. The above attributes prove the potential of lecithin as a candidate molecule for the prevention and therapy of cardiovascular diseases (Nowacki et al., 2017). Interestingly, eggs have been used in decoctions for medicinal plant extracts primarily to obtain food and health benefits in some ethnomedicinal practices (Xin et al., 2014; Wang et al., 2017).

## Role of Egg Components in Veterinary Medicine

### Effective Cryopreservation of Spermatozoa

Cryopreservation of veterinary spermatozoa can improve the mobility and stability of sperm as well as prevent loss of activity upon continuous freeze-thawing for prolonged durations (Kampschmidt et al., 1953). Replacement of the whole yolk with low-density lipoproteins (LDL) in extenders has gained much attention in recent years, providing higher plasma membrane integrity (Table 2). However, it has been noticed that the cryoprotective property of LDL greatly depends on the sperm characteristics obtained from different animal species as well as the percentage of LDL used to effectively replace whole egg yolk in conventional sperm preservation methods. Commercially available ready-to-use extenders with predefined standardized LDL concentrations specific to different animal species can be manufactured to improve semen quality and ease of use for consumers.

**TABLE 2:** Use of Egg yolk LDL in cryopreservation of animal spermatozoa.

Animal species	Extender used	Functional property	Reference
Canine Epididymal Spermatozoa	4% LDL in the extender	Post-thaw sperm motility, plasma membrane integrity, and acrosome integrity (57.69 ± 5.63, 70.54 ± 12.84, and 46.58 ± 10.79, respectively)	Prapaiwan et al. (2016)
Collared peccary (*Pecari tajacu*)	20% LDL in the extender	36.4 ± 5.3% higher post-thaw sperm motility, 27.4 ± 6.5% higher membrane-intact frozen-thawed spermatozoa	Souza et al. (2015)
Rabbit semen	10% LDL in the extender with sucrose	Improved the fertilization potential of the sperm, lower conception, and prolificacy rates	Iaffaldano et al. (2014)
Equine semen	2%–3% LDL supplemented with 2.5% glycerol in the extender	Good post-thaw sperm quality, improved spermatozoa motility	Moreno et al. (2013)
Rhesus monkey sperm	6%–10% of LDL with glycerol in extenders	Similar post-thaw motility to 20% whole egg yolk supplemented with glycerol	Dong et al. (2011)
Bull semen	8% LDL in extender	Improved post-thaw sperm motility, acrosome integrity, membrane integrity, highest antioxidant activities of CAT, GSH-Px, and GSH, reduced negative ROS impact on semen	Hu et al. (2011)
Buffalo bull semen	10% LDL on extender	Improved freezability, post-thaw motility, and fertility	Akhter et al. (2011)

### Poultry Feed Supplements

ESM contains numerous proteins and peptides, including collagens, laminin, agrin, keratin, clusterin peptides, and defensins, which have cryoprotective and chaperone-like functions as well as antimicrobial and immunomodulatory properties that are crucial for embryo development. ESM has emerged as an efficient feed supplement to post-hatchery chicks to improve immunity and egg-laying performance (Makkar et al., 2015). The protection of embryos upon treatment with ESM is attributed to the antimicrobial nature of lysozyme, keratin peptides, ovocalyxin, ovotransferrin, ovalbumin Y, ovostatin, ovomucoid, and ovoglycan, since a bacterial LPS-induced inflammatory response and a reduced level of stress markers such as plasma corticosterone levels were transferred to feed chicks (Rath et al., 2016). ES powder, a rich source of calcium, can improve the laying performance of birds (Cordeiro and Hincke, 2011). Consequently, ESM and ES obtained as discarded products from industrial egg waste can be recycled in a more efficient way to improve the quality of chicks from poultry, in a cyclic process that can be adopted by poultry manufacturing sectors to obtain diverse products with health benefits.

## Novel Alternative Drug Delivery Systems

### Ovomucoid as an Alternative Drug Delivery System

Existing drug delivery systems can be improved for more effective drug release at targeted sites with fewer side effects by using biocompatible materials such as proteins, nanoparticles, chitosan, and alginate. The mucoadhesive property of OVM can be exploited for the sustained release of drugs in the biological system through the mucus layer, which highly depends on mucus pH, viscoelasticity, mucin-to-water ratio, ion content, and turnover time of the luminous and adherent mucus layers (Abeyrathne et al., 2015). A drug delivery system containing OVM should be dealt with more specifically because of its ability to release drugs before replacement with a new mucous layer because the turnover time differs in each biological system.

The drug release kinetics of riboflavin, brilliant blue, and ciprofloxacin loaded onto OVM particles suspended in PBS, simulated intestine tract fluid (SIF), and simulated gastric tract fluid (SGF) showed that negatively charged ovomucin particles were better than the usual chitosan, alginate, and polyacrylic acid in establishing maximum detachment force and maximum viscoelasticity properties. Previous evidence showed that the system was suitable for heat-labile drugs because OVM is water-soluble and that the drawback of OVM degradation in saline environments can be rectified by the preparation of polyelectrolyte hydrogels to serve as the carrier for heat-labile pharmaceuticals (Akbari and Wu, 2017). In addition, cross-linked nanogels of egg yolk LDL prepared with N-hydroxysuccinimide proved to be an effective carrier for the delivery of curcumin encapsulated into nanogels, presenting good stability in both fasting and fed gastrointestinal conditions against digestive enzymes and aggregation under acidic conditions. Cross-linking provided a sustained slow release of curcumin from the nanogel with better performance than non-cross-linked ones (Zhou et al., 2018).

### Lecithin Organogels for Applications in Topical Drug Delivery

Wrinkles, uneven skin tone, black spots, lines, and pigmentation are symptoms of skin aging that result from environmental pollution, poor skincare, and exposure to ultraviolet light. Cosmeceutical drug preparations can delay the process of aging and reduce skin damage, but a better delivery system is needed to penetrate the dermal layer more effectively. Lecithin organogels and phospholipids, in conjunction with suitable additives, are promising topical drug delivery vehicles (Date et al., 2011; Elnaggar et al., 2014). Lecithin from purified egg yolk is superior to other topical drug delivery systems by serving as an organic medium to improve the penetration of less permeable drugs into the dermal layer of the skin. The hydrophilic and lipophilic properties of lecithin make it a suitable carrier for most anti-aging drugs, and its natural biocompatibility makes it a safe drug delivery tool for use in products in the skin cosmeceutical industry (Raut et al., 2012).

## Biomaterial Fabrication in Tissue Engineering

### Albumin in Bioactive Glasses in the Grafting Process

Bioactive glass, which is composed of SiO_2_-CaO-MgO-P_2_O_5_ (Kokubo 1990) (97), is an important class of bioceramics (Peitl et al., 2012) and is used as a graft material to aid in new bone formation.

Albumin, the key protein in the human extracellular protein matrix, is involved in bioactive glass adsorption with implants as it surrounds the foreign bodies exposed in the bloodstream. The bioactive glass system SiO_2_-CaO-MgO-P_2_O_5_ immersed in simulated human plasma with calcium-phosphate (Ca-P) with or without albumin during a 7- and 14-day immersion period, as studied *via* XRD and AFM analyses, reported the formation of amorphous octa-Ca-P precipitates on the surface of albumin. On the other hand, a thin, easily detachable layer was observed in the glass immersed in albumin-free SBF, suggesting that post-adsorption of albumin to the bioactive glass surface acts as a bridge between Ca^2+^ and PO^3-^ ions, thus forming a connection between the glass and the Ca-P layer (Paiva et al., 2007; Peitl et al., 2012). The bone and tissue grafting process can be made feasible by the inclusion of albumin to stabilize the connection and support the stability of the layer of new bone tissues formed.

### Applications of Advanced Materials Design for Tissue Engineering

The highly crisscrossed linkage of protein fibers in the ESM, similar to egg white albumin, initiates the biomineralization of eggshell formation and plays a major role in protecting the developing embryo from microbial pathogens. ESM is often disposed of as industrial waste from egg processing factories despite recent attention towards the isolation of bioactive compounds from it (Intharapat et al., 2012; Baláž, 2014). Because of their ultrastructure, biomineralization in organisms proceeds through the properties of organic–inorganic composites, which can retain water and their intrinsic biodegradability, as well as microcapsules that facilitate nutrient delivery (Cordeiro and Hincke, 2011; Guru and Dash, 2014). The presence of collagen, hyaluronic acid, fibronectin, osteopontin, and calcium carbonate in the ESM makes it a template for cells in tissue as stable mechanical support to adsorb and interact with growth factors and signaling molecules to form fully functional tissues during regeneration of skin, bone, cartilage, and nerves. Therefore, ESM serves as a potential biomaterial for various tissue engineering applications (Sah and Rath, 2016).

OVM hydrogels have been prepared previously, utilizing their unique foaming property in preparing scaffolds of various porosities, along with gelatin, to maintain their pore size and stability (Carpena et al., 2017). OVM scaffolds subcutaneously implanted in rats showed good adhesion of rat bone marrow mesenchymal stem cells with a lower incidence of fibrosis, vascularization, and activation of alternate macrophages. In addition, there is evidence for the satisfactory formation of blood vessels in mesenchymal stem cells with minimal macrophage infiltration and hence eliciting a weaker immunological response.

A biocompatible, non-toxic material with high elasticity, tensile strength, and tunable mechanical properties can be used for the fabrication of scaffolds in tissue engineering (You et al., 2017). The preparation of implants, scaffolds, and hydrogels with bioactive compounds from egg sources possessing tunable properties with enhanced tissue regeneration activity shows promise for tissue engineering applications.

## Development of Biosensors and Detection Systems

### ESM in Electrical Devices and Engineering Applications

The highly unique crisscrossed micro- and nanostructures of ESM make it a functional platform for the preparation and fabrication of materials in electrical devices, including sensors for environmental monitoring and biomedical engineering applications (Park and Kim, 2017). Given that the carbonized ESM has a good electric capacity, the adsorption property of the carbonized ESM can effectively allow the removal of on-site pollutants using fluorescent dyes (Shao et al., 2011; Xiong et al., 2012). Both native and functionalized forms of ESM have been used to develop biosensors for the detection of specific biomolecules from different samples (Ray and Roy, 2016). High amounts of amino acids and glycoproteins present in the ES and ESM make them efficient in cell adhesion and viability for applications in biomedical engineering. Since the commercial availability of ESM is currently very limited, it can be procured as supplementary material for the further study of shell membranes.

### Alternative to Antibodies in Toxin Detection Systems

The development of a highly accurate, rapid, and sensitive toxin detection platform is crucial for monitoring the consequences of the release of harmful toxins by microbial pathogens. Lectin is a biomolecule found in both plants and animals and plays a dual role with beneficial and harmful benefits when used as a biological sensor. Lectins possess a high affinity towards synthetic glycan ligands and antibodies that are currently employed in available toxin detection systems. Affinity nanoprobes functionalized with OVA with higher sensitivity and specificity for the detection of multiple lectins (ConA, BanLec, and ricin) in a single system have been developed (Selvaprakash and Chen, 2018). Lectin interactions with glycan ligands can be selectively released from nanoprobes by adding sugars such as mannose, glucose, and β-galactose as releasing agents. The replacement of antibodies with OVA probes for multi-lectins proves to be a cost-effective alternative with much higher sensitivity and specificity for the molecules of interest. Antibodies from mammalian sources can be avoided to an extent by replacing them with egg OVA in toxin detection systems, thus favoring the manufacture of portable kits for applications that need rapid processing.

### IgY Antibodies in Immunoassays

The presence of trace amounts of microbial toxins, hazardous chemicals, pollutants, toxic metals, and xenobiotic compounds in the environment and food samples is a major concern to human health, as prolonged exposure results in devastating long-term health issues. The presence of antibiotics poses a threat to multidrug-resistant microbial strains and superbugs. Minute levels of antibiotic residues in complex matrices raise the challenge of developing an appropriate analytical method for detection. IgY antibody-based enzyme-linked immunosorbent assays have been developed for the detection of antibiotics such as kanamycin and gentamicin from animal-derived food samples (Li et al., 2016). A similar antibody immunoassay platform was developed for the screening of a vital enzyme, CYP2E1 inhibitor/enhancer, from herbal medicines (Jiang et al., 2016). This approach made IgY-based systems detect microbial toxins, hazardous chemicals, pollutants, toxic metals, and foreign biological compounds in a wide variety of samples.

## Egg Components on Industrial Applications

### Latent Source of Keratin Sulfate

Corneal keratin sulfate plays a major role in ocular inflammation, corneal injury, and keratitis, and is a major component of ocular medications to restore normal visual conditions (Pomin, 2015). Cartilage keratin sulfate (KS) maintains the hydration of tissues, making them resistant to physical stress (Hayashi et al., 2011). Fully sulfated KS disaccharide has been proposed as a potent drug for the treatment of chronic obstructive pulmonary disease (Shirato et al., 2013). KS deficiency is attributed to the early-phase pathogenesis of amyotrophic lateral sclerosis, making it a possible therapeutic agent for amyotrophic lateral sclerosis (ALS) (Hirano et al., 2013). Previously, a highly potent KS molecule was prepared from bovine corneas but was eventually withdrawn due to an outbreak of bovine spongiform encephalopathy. The pharmaceutical industry has explored the potential of KS to increase demand and identified EW as a potential source of keratin sulfate, hyaluronic acid, chondroitin sulfate, and heparin sulfate for pharmaceutical preparations (Fu et al., 2016).

### Industrial Role of Lecithin and Phospholipids

Phospholipids in eggs can improve serum lipid metabolism, prevent aging and arteriosclerosis, and improve lipid metabolism in the liver. In addition, these molecules can promote the absorption of fat-soluble vitamins by inhibiting cholesterol and neutralizing fat, which can be used as parenteral lipid emulsions and drug delivery systems (Hartmann and Wilhelmson, 2001; Sahebkar, 2013; Blesso, 2015). The pharmaceutical industry uses phospholipids as wetting agents, emulsifiers, and builders or components of mesophases, such as liposomes, micelles, and mixed micelles (van Hoogevest and Wendel, 2014). Lecithin, a mixture of phospholipids, is mainly extracted from eggs and vegetables, including soybeans, sunflower, rapeseed, and cottonseed. As a natural emulsifier, lecithin can be applied in the food industry at an estimated world market demand of 150,000–170,000 tons, next to its applications as cosmetics and lubricants. Its molecular structure, with dual hydrophilic and lipophilic groups, makes it a surface-active molecule contributing to emulsification, anti-spattering, wetting, anti-staling, dough-conditioning, and antioxidant functions in various foods. Hence, lecithin has emerged as an important ingredient in food products because of its emulsifying properties (Cui and Decker, 2016).

Lecitihin, a polyunsaturated phosphatidylcholine that is a functional and structural component of all biological membranes, acts as the rate-limiting step in the activation of membrane enzymes such as superoxide dismutase (Hanin et al., 1990). Ineffective activation of these antioxidant enzymes leads to increased damage to membranes by reactive oxygen species (Hanin et al., 1990). In addition, lecithin increases bile secretion, prevents stagnation in the bladder, and consequently decreases lithogenicity (Herron and Fernandez, 2004; Miranda et al., 2015). It also acts as an emulsifier in enteral formulas to reduce the incidence of diarrhea in rats (Akashi et al., 2017). Formulations containing egg yolk lecithin, medium-chain triglycerides, and dietary fiber for their potential use in lipid absorption have also been studied in rats with short bowel syndrome. The results showed no difference in the average particle size of egg yolk lecithin emulsifier upon the addition of artificial gastric juice, increased serum triglyceride concentrations, and improvement in fecal consistency and bowel movement frequency. The enteral formula promoted lipid adsorption by preventing the destruction of emulsified substances by gastric acid and improving diarrhea. Replacing conventional oral formula with lecithin as an emulsifier may reduce the incidence of diarrhea, thereby reducing medical costs due to hospitalizations.

## Egg Components in Various Industrial Sectors

### ES and ESM as Emerging Vital Constituents in Multiple Industries

In addition to the efficient capability of ES waste to eliminate dyes from industrial effluents, its notable roles in other industries extend to its use as a catalyst in industrial processes such as biodiesel synthesis, synthesis of dimethyl carbonate (DMC), synthesis of hydrogen/syngas, wastewater treatment plants, and pollutant removal or immobilization in liquid, soil, or gaseous emissions (Quina et al., 2016; Laca et al., 2017). Interestingly, calcium carbonate purified from ES waste has been recently utilized as a building material along with cement and mortar, enhancing the strength of buildings and other improvements in the construction industry. The brightness and smoothness of writing materials such as papers can be improved by including ESW in the paper manufacturing process, as well as in the paint and dye manufacturing industries (Kirboga and Öner, 2013). Both ES and ESM are used as substrates for the enzymatic production of alkaline protease from *Bacillus altitudinis* GVC1 along with maltose as an additional carbon source, increasing the enzyme production by up to 13%, proving their potential in increasing production capacity in microbial enzyme manufacturing industries (Nagamalli et al., 2017).

### Egg Components in Cosmeceutical Preparations

ES and ESM proteins are arranged in a perfect matrix by the fibrous protein collagen and play a crucial role in tissue structures by supporting them in place between the shell and the membrane. This property of collagen is exploited in preparing skincare cosmeceutical products to foil skin wrinkles and to improve the elasticity and thickness of the skin (Cordeiro and Hincke, 2011). Cosmeceutical preparations containing natural ingredients such as lecithin and phospholipids as a substitute for synthetic emulsifiers and functional additives helps fulfill the growing demand for natural cosmetic formulations. Lecithin and phospholipids obtained from eggs possess excellent hydration, soothing properties, and applications in cosmetic creams, lotions, and gels, providing satisfactory moisturizing effects with negligible irritability as they are biocompatible to human skin.

## Role of Egg Components in Environmental Protection

### Elimination of Toxic Chemicals From Water Resources

Most populations generate tons of ES waste; industries also emit huge volumes of industrial effluents into water bodies despite strict regulations, drastically affecting the food chain. Both issues involve environmental protection and can be solved using egg-based products. In addition to materials such as peat, plant by-products, and activated charcoal used for the removal of toxic chemicals, ES can also adsorb organic industrial dyes and pigments, such as direct red 80, acid blue 25, methylene blue, brilliant green, and malachite green (Podstawczyk et al., 2014) from textile industrial effluents. The biosorption mechanism of harmful textile dyes by ES lies in the physical adsorption of the dyes to the cell wall of dead cells and the associated functional groups that constitute the ES. This, combined with micro-precipitation, reduces the concentration of chlorinated phenols, fluorides, phosphate, and other organic pollutants in water bodies worldwide (Guru and Dash, 2014; Quina et al., 2016). The presence of calcium carbonate and the protein acid mucopolysaccharides with distinguished pore structures in the shell aid the development of nanomaterials for the removal of toxic heavy metals, including lead, copper, and cadmium, from the environment (Chumlong et al., 2007). Thus, large quantities of ESW can be utilized in a more appropriate way to save organisms thriving in water bodies by eliminating toxic chemicals and industrial dyes.

### Enriching Plant Growth and Development

Soil nutrient availability, along with the physical parameters (pH and salinity), has a great influence on plant growth. When soil pH decreases and becomes highly acidic (pH < 4.5), a decline in calcium carbonate level occurs, leading to a loss of bioaccessibility to the plant, interfering with its growth and development. The addition of ESW as compost or co-compost material has been shown to reverse the calcium carbonate loss, thereby increasing the capacity of the soil to improve the growth and yield of plants (Soares et al., 2015). Compost materials, including ESW, can be manufactured to enhance horticulture activities more efficiently.

## Next-Generation Transgenic and Enriched Eggs

**FIGURE 4:**
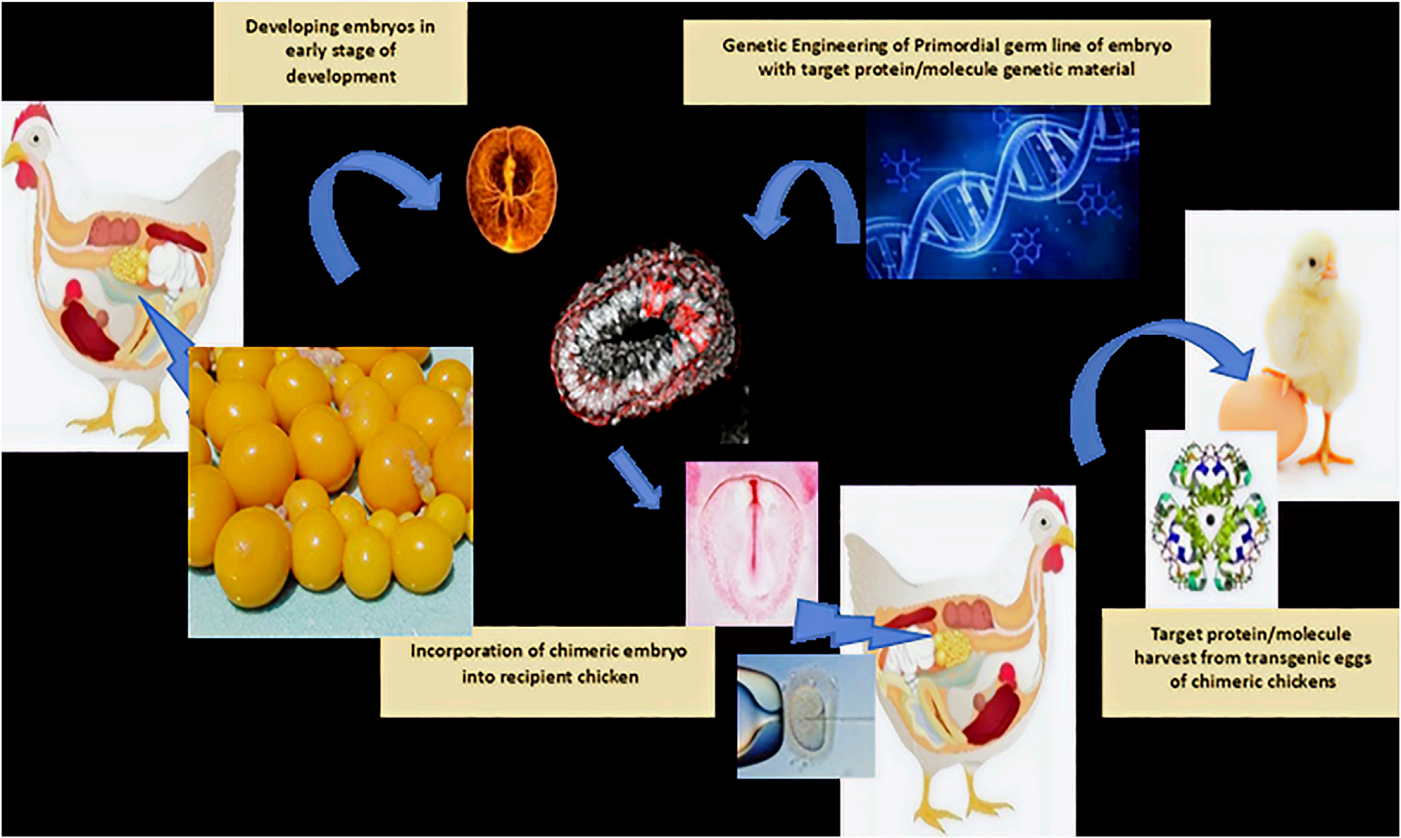
Next-generation customized chickens through transgenesis.

Eggs can be enriched with specific molecules, such as omega-3 fatty acids, vitamins, minerals, docosahexaenoic acid (DHA), selenium, to have lower cholesterol, or become pigmented eggs, among others (Laudadio et al., 2015; Singh et al., 2012). Herb-enriched eggs have been developed by feeding hens with herbs containing active compounds such as allicin, betaine, eugenol, lumiflavin, lutein, taurine, and sulforaphane, which showed a significant reduction in triglyceride levels, increased levels of HDL cholesterol, improved immunity, and increased hematocrit levels, as tested in human volunteers (Zaheer, 2015). Furthermore, functionally modified eggs through genetic manipulation of the chicken immune system to produce a specific protein, peptide, or molecule of pharmaceutical interest can allow its natural retention in the system and later harvest, such as insulin molecules or antibody-enriched eggs (Figure 4) (Sang, 1994; June Byun et al., 2017; Bednarczyk et al., 2018; Ching et al., 2018).

Despite the identification of novel peptides with unique biomedical properties, large-scale production is still being continuously improved to achieve a sustainable turnover for industries. Industrial-scale production of bioactive peptides is feasible with different techniques involving supercritical fluid technology, magnetic particles for separation of specific egg compounds by immune magnetic separation (Huopalahti, 2007), and enzymatic hydrolysis of egg white albumin and yolk followed by ultrafiltration to obtain low-molecular-weight peptides combined with chromatographic techniques (Nimalaratne et al., 2015). Egg components, including lysozyme, avidin, and IgY antibodies, are currently under industrial production using standardized purification protocols that can be extended to identify other bioactive peptides.

### Summary and Future Prospects

Various egg components, including lysozyme, avidin, IgY, lecithin, and bioactive peptides, display anti-cancer, antihypertensive, anti-inflammatory, and antimicrobial activities, and have great industrial opportunities in the pharmaceutical sector. In recent years, the cosmeceutical industry has taken advantage of the beneficial effects of egg yolk lecithin to expand its use in many skincare products. Meanwhile, the wastes generated from the egg processing industries can be more efficiently utilized by other textile and dye manufacturing industries. For instance, recycling egg waste for the removal of toxic pollutants from industrial effluents is a synergistic process between the two industries, decreasing the environmental impact of the waste generated. The future of the egg industry lies in generating functional eggs by enriching them with specific compounds or preparing transgenic eggs *via* genetic manipulation of chickens to fulfill the need for the production of specific proteins in the eggs to treat various diseases. Recent advancements in the large-scale purification of compounds from eggs using newer methods such as supercritical technology and the separation of bioactive peptides *via* magnetic separation made it feasible for industrial preparations. Eggs harboring biologically and industrially important peptides encourage the exploration for other efficient bioactive peptides in the future, not only by the scientific community but also by the industrial sector.

## Data Availability

The original contributions presented in the study are included in the article/Supplementary Material. Further inquiries can be directed to the corresponding author.
